# Anti-Inflammatory and Cytostatic Activities of a Parthenolide-Like Sesquiterpene Lactone from *Cota palaestina* subsp. *syriaca*

**DOI:** 10.1155/2015/474597

**Published:** 2015-05-19

**Authors:** Rabih S. Talhouk, Bilal Nasr, Mohamed-Bilal Fares, Bushra Ajeeb, Rana Nahhas, Lamis Al Aaraj, Salma N. Talhouk, Tarek H. Ghaddar, Najat A. Saliba

**Affiliations:** ^1^Department of Biology, Faculty of Arts and Sciences, American University of Beirut, Beirut 1107 2020, Lebanon; ^2^Nature Conservation Center (NCC), American University of Beirut, Beirut 1107 2020, Lebanon; ^3^Brain Mind Institute, School of Life Sciences, Ecole Polytechnique Fédérale de Lausanne (EPFL), 1015 Lausanne, Switzerland; ^4^Department of Chemistry, Faculty of Arts and Sciences, American University of Beirut, Beirut 1107 2020, Lebanon; ^5^Landscape Design and Ecosystem Management, Faculty of Agriculture and Food Sciences, American University of Beirut, Beirut 1107 2020, Lebanon

## Abstract

A sesquiterpene lactone 1-*β*,10-Epoxy-6-hydroxy-1,10H-inunolide (K100) was isolated through “bioassay-guided fractionation” from *Cota palaestina* subsp. *syriaca*, an Eastern Mediterranean endemic plant. K100 inhibited endotoxin- (ET-) induced proinflammatory markers: IL-6, MMP-9, and NO in normal mouse mammary SCp2 Cells. Molecular docking *in silico* suggested that K100, having highly analogous structure as parthenolide (PTL), an anticancer compound, could bind PTL target proteins at similar positions and with comparable binding affinities. Both compounds, K100 and PTL, inhibited the proliferation and prolonged the S-phase of the cell cycle of breast adenocarcinoma MDA-MB-231 cells grown in 2D cultures. Noncytotoxic concentrations of K100 and PTL decreased the proliferation rate of MDA-MB-231 and shifted their morphology from stellate to spherical colonies in 3D cultures. This was accompanied with a significant increase in the amount of small colonies and a decrease in the amount of large colonies. Moreover, K100 and PTL decreased cellular motility and invasiveness of MDA-MB-231 cells. In summary, these results suggest that K100 exhibits PTL-analogous anti-inflammatory, cytostatic, and antimetastatic effects.

## 1. Introduction

*Cota palaestina* DC, a plant endemic to Lebanon known in Arabic as “Bahar ghishai,” belongs to the Anthemideae tribe of the Asteraceae (Compositae) family, which comprises 25,000 species within three subfamilies and 17 tribes [1], many of which are employed in a variety of medicinal applications. The plant is distributed between Lebanon (Anti-Lebanon and Beirut) and Syria (Aleppo, Wâdi-Barada, Jabal Zabad, Damascus, and Dummar) [2]. Over the centuries,* Anthemis* spp. were widely exploited in folk medicine due to the therapeutic effects they exhibited, including sedative, anti-inflammatory, antimutagenic, antigenotoxic, antiphlogistic, antiepileptic, mitogenic, spasmolytic, anxiolytic, antimycobacterial, insecticidal, and antimicrobial effects [3].

The chemical composition and biological activities of* Cota palaestina* remain poorly investigated. The water extract of its aerial parts was previously screened for antimicrobial [4] and anti-inflammatory activities [5, 6] showing efficacious inhibitory effects in both, yet its key chemical active components were not identified. Most* Anthemis* spp. that have been studied are known to contain sesquiterpene lactones (SLs), the most studied class of secondary metabolites [1, 7, 8]. So far, almost 5000 structures of SLs have been recorded in the Dictionary of Natural Products [9], of which more than 4000 with 30 different skeletal types have been reported within Asteraceae alone [1]. In the presence of an exocyclic *α*-methylene-*γ*-lactone moiety, these compounds are known to react by Michael-type addition with exposed cysteine residues in proteins, thereby inhibiting major cellular functions [10–14]. For instance, the extensively studied SL extracted from the plant* T. parthenium* “PTL” has been reported to exhibit antiproliferative activities upon binding and inhibiting the function of members of the NF*κ*B, JAK-STAT, and TNF*α* signaling pathways [15]. Moreover, a more soluble PTL analogue “di-methyl-amino-PTL (DMAPTL)” was shown to exhibit similar effects* in vivo* and has been tested in clinical trials [16].

Through bioactivity-guided fractionation of the water extract of* Cota palaestina*, this study attributes anti-inflammatory and cytostatic activities of this plant to the parthenolide like sesquiterpene lactone 1-*β*,10-*α*-Epoxy-6*α*-hydroxy-1,10H-inunolide (referred to hereafter as K100). This compound reduces inflammatory mediators interleukin-6 (IL-6), nitric oxide (NO), and matrix metalloproteinase-9 (MMP-9) production by endotoxin- (ET-) treated mammary epithelial cells (SCp2), a culture system previously reported as effective in screening for anti-inflammatory bioactive molecules [17]. The cytostatic activity is attributed to the ability of K100, similarly to PTL, to partially revert growth phenotype, invasion, and motility of MDA-MB-231 breast adenocarcinoma cells.

## 2. Results and Discussion

### 2.1. Plant Identification

**Figure 1:**
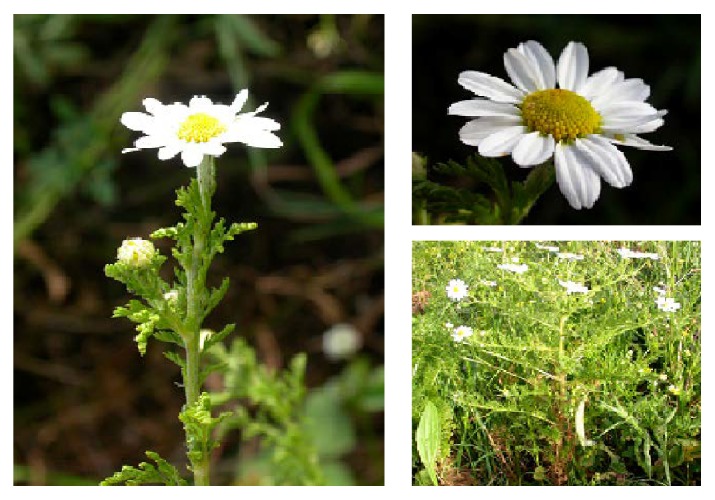
Plant identification: photomicrographs of* Cota palaestina* showing a specimen that is deposited at the herbarium of the American University of Beirut, Beirut, Lebanon (photos courtesy of Khaled Sleem 2008, Landscape Design and Ecosystem Management, Faculty of Agriculture and Food Science, American University of Beirut, Beirut, Lebanon). From Flora of Syria, Palestine, and Sinai [2].

In this study, we report, for the first time, the existence of anti-inflammatory and antitumor activities in* Cota palaestina* Kotschy [*palaestina* subsp.* syriaca* (Bornm.)* R. Fern.*], an Eastern Mediterranean plant endemic to Lebanon and known in Arabic as “Bahar ghishai.”* Anthemis scariosa*, now known as* Cota palaestina* spp.* syriaca* after the recognition of* Cota* as an independent genus [18–20], was selected for further study among 27 other indigenous Lebanese wild plant species that have been commonly used in Lebanese folk medicine [5]. The plant grows in Dayr-ul-Ahmar to Aynata region at elevations of 1200–1800 m above sea level, respectively. It is also reported in Anti-Lebanon Mountain range above Ayn-Burday at 1250–1300 m. The plant (Figure 1) is glabrous and is branching from neck; its stems are erect and are sparingly branched. The leaves are oblong in outline; its peduncles are long not thickened. The flowering season is during the months of April and May [2].

### 2.2. Identification and Structural Elucidation of Anti-Inflammatory Component “K100” in* Cota palaestina*

**Figure 2:**
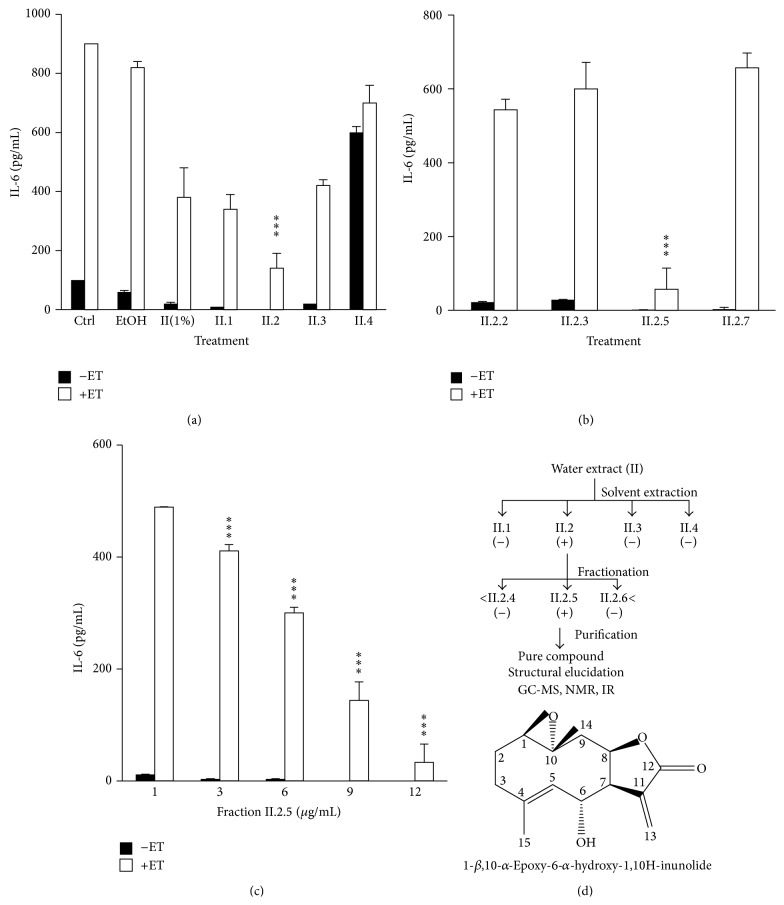
Identification of the anti-inflammatory component “K100” in* Cota palaestina*: (a) effect of II.1, II.2, II.3, and II.4 fractions (10 *μ*g/mL). (b) Effect of subfractions (II.2.2, II.2.3, II.2.5, and II.2.7) at 10 *μ*g/mL on IL-6 production and (c) dose-dependent inhibition of IL-6 production by fraction II.2.5 at 1, 3, 6, 9, and 12 *μ*g/mL. (d) Purification and structure of the anti-inflammatory component “K100” in* Cota palaestina*. Diagram summarizing the fractionation of* Cota palaestina* extract. (−) indicates no suppression of IL-6 production by ET-treated SCp2 cells and (+) indicates suppression of IL-6 production. Chemical structure of K100, the germacranolide 1-*β*,10-*α*-Epoxy-6-hydroxy-1,10H-inunolide. Statistical significance is represented by (*∗∗∗*) asterisk indicating significant difference at *p* < 0.001.

The capacity of the decoction and subsequent fractions of the aerial parts of* Cota palaestina* to modulate IL-6 levels produced by ET-treated SCp2 cells was evaluated by quantifying levels of IL-6 at 9 h following ET treatment. Among the fractions (II.1, II.2, II.3, and II.4 at 10 *μ*g/mL), only fraction II.2 suppressed ET-induced IL-6 levels beyond that noted for 1% decoction (Figure 2(a)). Interestingly, fraction II.4 enhanced IL-6 production in non-ET-treated cells, suggesting a proinflammatory bioactivity not noted in whole extracts of the plant. Upon further fractionation of II.2 by column chromatography, fraction II.2.5 was found to be the most active among the fractions (II.2.2, II.2.3, II.2.5, and II.2.7) at 10 *μ*g/mL (Figure 2(b)). The inhibition of IL-6 production by this fraction was concentration-dependent (Figure 2(c)). This fraction was further purified (Figure 2(d)) to yield a pure compound (II.2.5.3).

Spectroscopic data confirmed that the germacranolide 1-*β*,10-*α*-Epoxy-6*α*-hydroxy-1,10H-inunolide (Figure 2(d)), previously identified in* Mikania goyazensis* [21], was isolated from* Cota palaestina* and reported here, for the first time, to possess an anti-inflammatory and cytostatic activity. The potency of this compound, K100, is primarily attributed to the *α*-methylene-*γ*-lactone moiety, which targets and inhibits the activity of several functional proteins. Moreover, several studies [10, 14, 22–26] have shown that additional functional groups neighboring the *α*-methylene-*γ*-lactone can enhance the activity of SLs. In the case of K100, the hydroxyl group at C6 does not seem to influence the activity of K100 higher than PTL. It is the epoxide moiety that seems to influence K100 and PTL in the same way, hence both exhibiting similar biological activities. One could also suggest that epoxide can participate in intermolecular hydrogen bonding with amino acid residues adjacent to the reactive center of the target protein [10].

### 2.3. K100 Inhibits IL-6, MMP-9, and NO Production in ET-Treated SCP2 Cells

**Figure 3:**
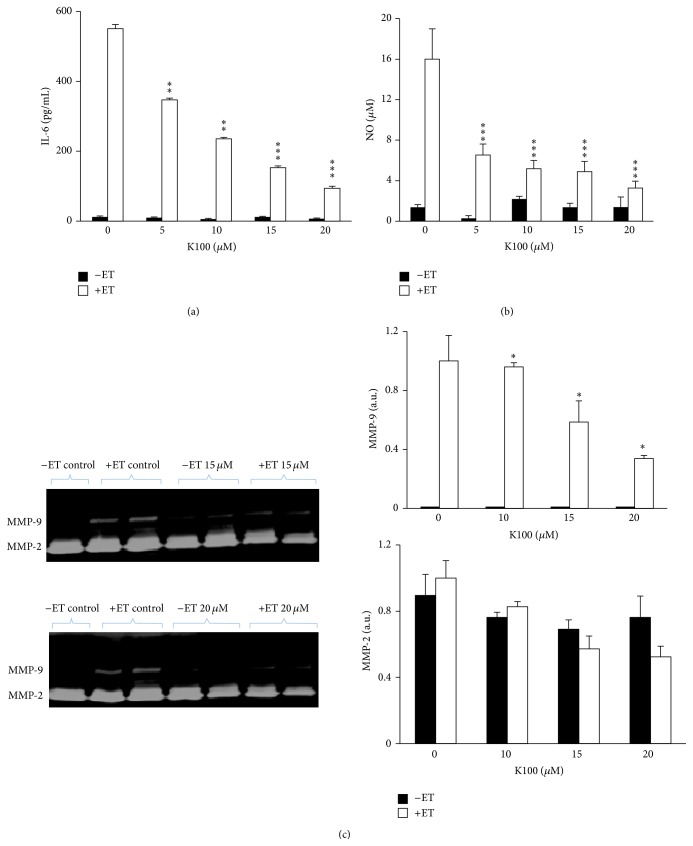
K100 inhibits IL-6, NO, and MMP-9 production in ET-treated SCp2 cells. Cells were treated with 0, 5, 10, 15, and 20 *μ*M of K100 and media samples were collected 24 hrs after ET-stimulation and analyzed for their (a) IL-6 secretion, (b) NO production, and (c) MMP-9 and MMP-2 production. Zymograms were analyzed by Gel documentation (Bio-Rad) using the software Quantity 1. Statistical significance is represented by (*∗∗∗*) asterisk indicating significant difference at *p* < 0.001, (*∗∗*) asterisk indicating significant difference at *p* < 0.01, and (*∗*) asterisk indicating significant difference at *p* < 0.05.

The pure compound K100 retained the anti-inflammatory activity and inhibited IL-6 production in ET-induced SCp2 cells [27] at noncytotoxic concentrations as determined by trypan blue exclusion assays and in a concentration-dependent manner, as shown in Figure 3(a). Interestingly, K100 inhibited NO production in ET-induced SCp2 cells in a dose-dependent manner with significant inhibition reaching 56% at a concentration as low as 5 *μ*M (Figure 3(b)).

Furthermore, SCp2 cells in culture produced abundant levels of MMP-2 (gelatinase A) in ET-treated and untreated cells; however MMP-9 (gelatinase B) was markedly upregulated in ET-treated cells. K100 inhibited, in a dose-dependent manner, MMP-9 in conditioned media at 24 hrs after ET treatment reaching a 40% decrease at 20 *μ*M. However, only partial inhibition of MMP-2 was observed with 15 and 20 *μ*M concentrations at 24 hrs after ET treatment (Figure 3(c)). In the three experiments K100 did not cause a significant change in the levels of IL-6, NO, and MMP-9 in control SCp2 cells not treated with ET (Figure 3). Several studies on plant extracts have shown an inhibitory effect for diverse plant secondary metabolites, namely, terpenoids and phenols against IL-6, NO, and MMPs production [28, 29]. However, the ability of K100 to inhibit ET-induced cytokine IL-6, MMP-9, and NO, all common mediators of cancer and inflammation [30] and markers of dedifferentiation [27, 28, 31], prompted further investigation into its structure and bioactivities.

### 2.4. *In Silico* Molecular Docking to Known Parthenolide (PTL) Targets Shows That K100 Exhibits PTL-Analogous Predicted Binding

**Figure 4:**
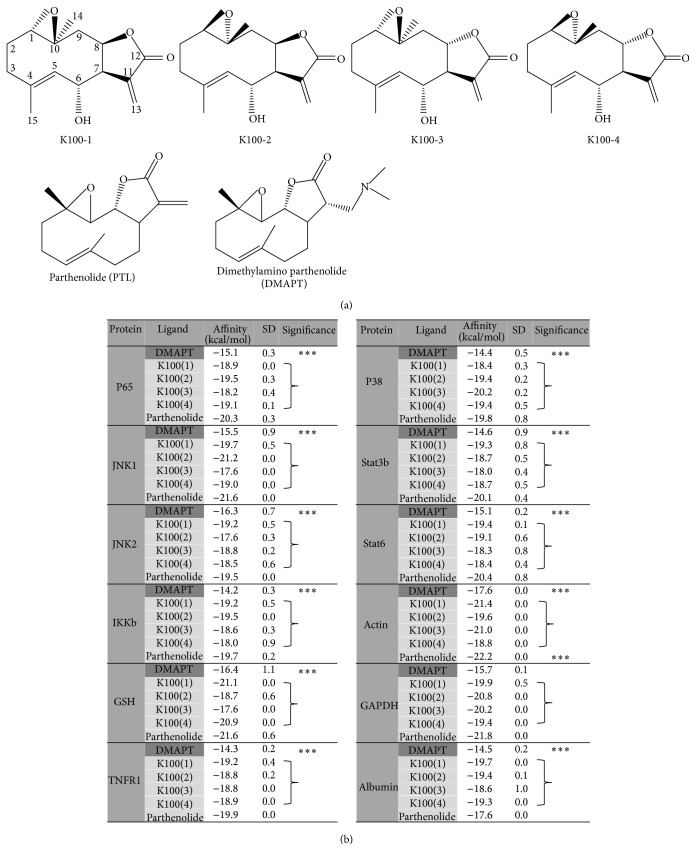
*In silico* molecular docking shows that K100 exhibits PTL-analogous predicted binding to known PTL targets: (a) chemical structure of the four K100 stereoisomers and the other two SLs included in the docking simulations, PTL and DMAPT. (b) Predicted binding affinities of K100 (1), K100 (2), K100 (3), K100 (4), PTL, and DMAPT to IKKb, p65, p38, JNK1, JNK2, Stat3b, Stat6, TNFR1, GSH, Actin, GAPDH, and Albumin. Numbers shown represent averages of 5 independent simulations ± standard deviation (SD). Statistical significance is represented by asterisk (*∗∗∗*) at *p* < 0.001.

K100 bears similar functional groups to those found in PTL, namely, a 6-6-5 ring germacranolide basic structure with an exocyclic *α*-methylene-*γ*-lactone moiety, an epoxide at C1, and a double bond at the C4 position. Structural analysis of K100 has revealed that this molecule has many possible stereoisomers; however, the most probable ones are K100-1 to 4 (Figure 4(a)) with different configurations at the three chiral centers C1, C10, and C8 positions [21, 32]. Docking analysis* in silico* to assess the ability of K100 to bind to PTL and DMAPT targets such as the TNF*α* receptor, IKKb, the NF*κ*B subunit P65, and members of the STAT signaling pathway [15] revealed that K100 was capable of binding to similar PTL protein targets with a comparable binding affinity (Figure 4(b)). In fact, the values showing the lowest docking energies that reflect the strongest binding affinities clearly indicate that the binding affinities of K100 to PTL targets are very close to those of PTL suggesting that the four K100 stereoisomers can bind to PTL protein targets with a similar affinity as that of PTL. Despite being predictive [33, 34], these results further confirm the structural homology between PTL and K100 and indicate that the hypothesized physiological effects of K100 could be exhibited via pathways similar to those utilized by PTL.

### 2.5. Effect of K100 and PTL on the Proliferation, Morphology, and Cell Cycle Progression of MDA-MB-231 Cells under 2D and 3D Culture Conditions

**Figure 5:**
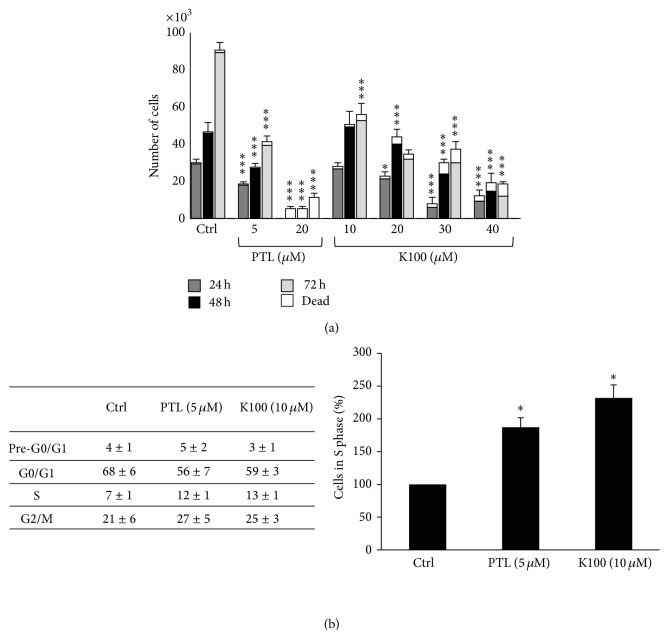
Effect of K100 versus PTL on the proliferation and cell cycle progression of MDA-MB-231 cells under 2D culture conditions: (a) effect of different concentrations of K100 versus PTL on MDA-MB-231 cell proliferation after treatment for 24, 48, and 72 hrs. Cellular viability was measured by trypan blue exclusion dye assay. (b) Table representing percentage of cells in G0/G1, S, and G2/M phases of cell cycle in control cells and cells treated with K100 versus PTL. Histogram plot showing cell counts in S-phase with respect to untreated cells. Statistical significance is represented by (*∗∗∗*) asterisk indicating significant difference at *p* < 0.001, and (*∗*) asterisk indicating significant difference at *p* < 0.05.

We next sought to establish whether K100 exhibits PTL-analogous antiproliferative effects using the breast MDA-MB-231 adenocarcinoma cell line as a model. K100, at noncytotoxic concentrations of 10 and 20 *μ*M, inhibited the growth of MDA-MB-231 cells in a dose-dependent manner (Figure 5(a)). PTL also inhibited the growth of MDA-MB-231 cells at 5 *μ*M but showed high toxicity at 20 *μ*M concentrations (Figure 5(a)). Interestingly when compared to PTL, K100 exhibited a larger differential between concentrations affecting cancerous but not normal cells (data not shown).

Given these results, we chose 10 *μ*M K100 and 5 *μ*M PTL as the effective noncytotoxic working concentrations throughout the rest of the study. The decrease in cell proliferation was accompanied by a 86% prolongation of S-phase of the cell cycle in cells treated with 10 *μ*M K100 compared to a 71% prolongation in cells treated with 5 *μ*M PTL (Figure 5(b)). On the other hand, no significant differences were noted in pre-G0/G1, G0/G1, and G2/M phases of the cell cycle when comparing cells treated with K100 versus PTL with untreated cells. Whereas no previous studies exist on K100, existing studies have shown that PTL is capable of suppressing the proliferation and inducing apoptosis in many human cancer cells* in vitro*, such as hepatoma, human lung carcinoma (A549), human medulloblastoma (TE671), cholangiocarcinoma, and colorectal and pancreatic cancers [15, 35–37]. Furthermore, other reports have shown that PTL can specifically eradicate malignant cells [16]. These studies showed that PTL preferentially targets leukemia stem cells while sparing normal hematopoietic stem cells.

**Figure 6:**
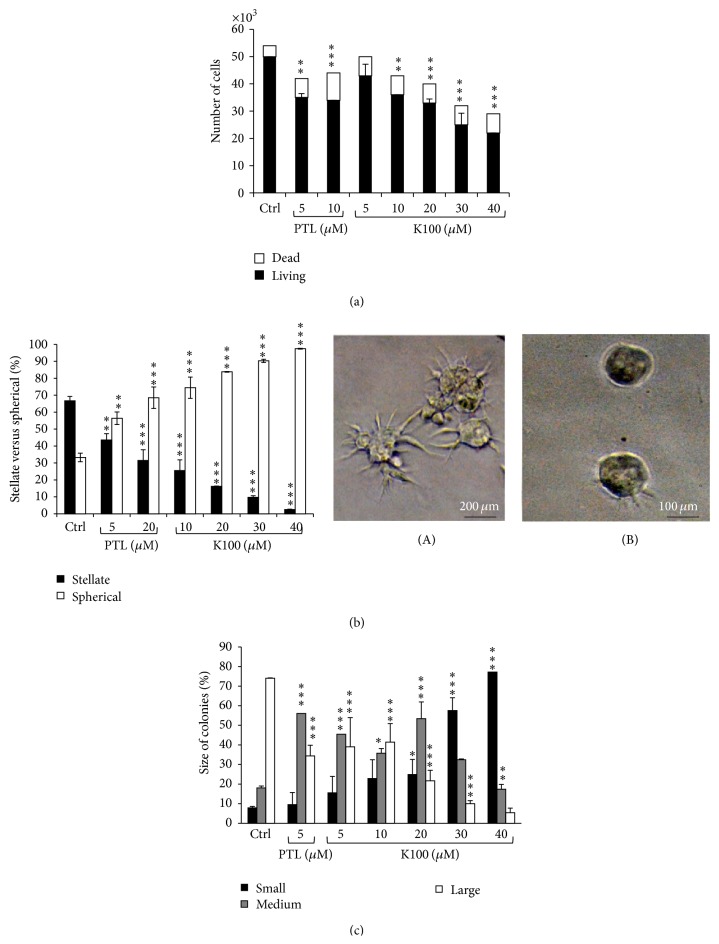
Effect of K100 versus PTL on the proliferation rate, morphology (% of stellate versus spherical colonies), and size of colonies in 3D culture: (a) effect of K100 versus PTL on the proliferation rate of MDA-MB-231 cells in 3D culture conditions on day 8 of culture. Bar graph showing number of untreated, K100-treated, and PTL-treated MDA-MB-231 cells. (b) Effect of K100 versus PTL on the morphology of MDA-MB-231 cells in 3D culture conditions after three days. Phase contrast photomicrographs of MDA-MB-231 cells showing stellate colonies (A) of untreated cells and spherical colonies (B) obtained upon treatment with K100 or PTL. Histogram showing percent stellate versus spherical colonies of untreated and K100- or PTL-treated MDA-MB-231 cells at day 3. (c) Histogram showing colony sizes analysis, after grouping colonies in 3D cultures into small (less than 70 *μ*m), medium (between 70 and 100 *μ*m), and large (greater than 100 *μ*m) clusters. Statistical significance is represented by (*∗∗∗*) asterisk indicating significant difference at *p* < 0.001, (*∗∗*) asterisk indicating significant difference at *p* < 0.01, and (*∗*) asterisk indicating significant difference at *p* < 0.05.

PTL has been reported to cause cell cycle progression arrest at the G2/M check point in the sarcomatoid hepatocellular carcinoma cell line (SH-J1) and at the G1 phase in the human 5637 bladder cancer cells and at G0/G1 phase in melanoma cancer cell lines [38, 39]. We also assessed the effect of K100 and PTL on the growth and proliferation of MDA-MB-231 cells cultured in 3D on growth factor reduced matrigel. There is now considerable interest in developing 3D* in vitro* systems for testing the efficacy of anticancer drugs [40]. Similar to 2D cultures, treatment of MDA-MB-231 cells with different concentrations of K100 versus PTL significantly decreased the growth rate of MDA-MB-231 cells grown in 3D cultures (Figure 6(a)). The number of cells treated with K100 was reduced in a dose-dependent manner starting with approximately 30% reduction at 10 *μ*M reaching more than 50% at 40 *μ*M with a cell death percentage not exceeding 14% in treated cells. Similarly, the growth of cells treated with PTL was reduced by approximately 30% with 20% cell death. It is worth noting that both K100 and PTL were shown to be cytotoxic in 2D cultures at concentrations greater than 10 and 20 *μ*M, respectively. Our findings are in line with previous studies showing that cultures in 3D matrices desensitize cancer cells to drug treatments [41]. Cells grown in 3D are more resistant to chemotherapy than those grown in 2D cultures and studies suggest important roles for cellular architecture, phenotypic variations, and extracellular matrix in drug transport and in drug efficacy [42]. Moreover, it has been shown that small molecules including drugs could bind to proteins found in matrigel such as collagen and laminin, thereby decreasing the effective concentrations reaching cells [43].

In 3D cultures, the majority of MDA-MB-231 untreated cells form stellate colonies, typical of these cells [44], upon adhering to the matrigel; however cells treated with K100 and PTL show an increase in the percentage of spherical colonies that resemble the growth of normal mammary epithelial cells on matrigel (Figure 6(b)). Counting stellate and spherical colonies on day 3 in 3D cultures showed that K100 increased the percentage of spherical colonies as compared to untreated cells in a dose-dependent manner. At 10 *μ*M, the number of spherical colonies increased to more than 70% and at 40 *μ*M up to 97% compared to less than 35% in control untreated cells. Similarly, treatment with PTL increased the percentage of spherical colonies as compared to untreated cells with approximately 55% and 70% at 5 *μ*M and 20 *μ*M PTL, respectively (Figure 6(b)).

Cluster size grouping, based on cluster diameter, showed that small sized clusters (diameter between 10 and 35 *μ*m) were abundant in cells treated with K100 reaching 77% of total clusters upon treatment with 40 *μ*M K100 compared to 7% in untreated cells. Moreover, the percentage of large sized clusters (diameter larger than 100 *μ*m) decreased in cells treated with K100 reaching 6% with 40 *μ*M K100 compared to 74% in untreated cells (Figure 6(c)). On the other hand, cells treated with 5 *μ*M PTL had the same percentage of small sized clusters (9%) as that of untreated cells (8%) but showed an increase in percentage of medium sized clusters (diameter between 70 and 100 *μ*m) and a decrease in large sized clusters as compared to untreated cells (Figure 6(c)). This switch in morphology has been observed in the poorly differentiated Hs578T breast cancer cell line when treated with geodiamolide H, a depsipeptide isolated from the marine sponge* Geodia corticostylifera* [45]. It was shown that the geodiamolide H was able to revert the stellate clustering of Hs578T cells plated on top of matrigel into spherical-like aggregates that resemble normal polarized acini of MCF-10A cells. Moreover, this reversion into normal-like spheroids was accompanied by an inhibition in cellular motility and invasion and was coupled with a partial acquisition of a polarized phenotype [46]. Essentially, our data indicates that the morphogenic reversion from stellate into spherical clustering suggests that K100 is able to partially revert the mesenchymal phenotype in MDA-MB-231 cells.

### 2.6. K100 Reduces Invasiveness and Motility of MDA-MB-231 Cells

**Figure 7:**
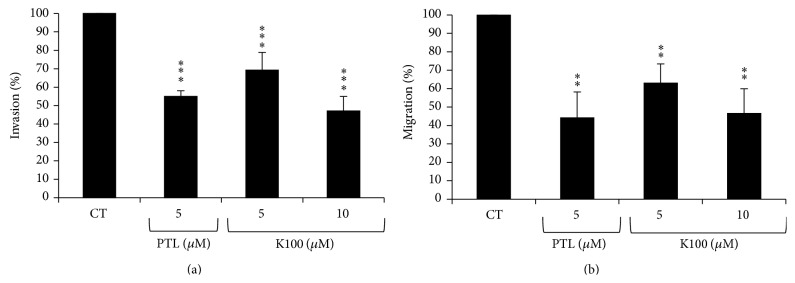
Effect of K100 versus PTL on the invasiveness and motility of MDA-MB-231 cells: (a) histogram showing percentage of untreated invading cells versus others treated with K100 versus PTL. (b) Histogram showing percentage of untreated migrating cells versus others treated with K100 or PTL. Statistical significance is represented by (*∗∗∗*) asterisk indicating significant difference at *p* < 0.001, (*∗∗*) asterisk indicating significant difference at *p* < 0.01, and (*∗*) asterisk indicating significant difference at *p* < 0.05.

Given that treatment with K100 and PTL induced spherical cluster formation of MDA-MB-231 cells in 3D cultures, suggesting that these cells might have a reduced ability to invade through matrigel, we sought to determine the effect of K100 and PTL on the transendothelial invasive ability of these cells. For that purpose, we used MDA-MB-231 calcein-labeled tumor cells or transfected with an EGFP (data not shown) which we previously demonstrated to have the same growth and invasive properties as control untransfected MDA-MB-231 cells [31]. Cell counting of extravasated cells after 16 hrs showed that cells treated with 5 or 10 *μ*M K100 versus 5 *μ*M PTL exhibited reduced invasive ability by 31%, 53%, and 45%, respectively (Figure 7(a)).

Similarly, transwell motility assays of MDA-MB-231 cells over transwell filters coated with growth factor reduced matrigel components (without endothelial cells) showed that 5 or 10 *μ*M K100 versus 5 *μ*M PTL decreased cellular motility by 37%, 53%, and 56%, respectively (Figure 7(b)).

PTL has been reported to inhibit the invasiveness and migration of BxPC-3 pancreatic cancer cells [47] and reduce the metastasis of MDA-MB-231 cells in a xenograft model of breast cancer in combination with docetaxel [48]. In other studies, PTL has been shown to have an antimigratory effect on breast cancer MCF-7 cells and displayed apoptosis-mediated cytotoxic effects against breast cancer MDA-MB-231 and MCF-7 adenocarcinoma cells [49, 50]. Moreover, it has been recently shown that PTL suppresses mRNA levels of MMP-9 and VEGF in (A375) melanoma cells [39] and osteosarcoma cell line LM8, respectively [51]. No similar studies are reported on K100.

## 3. Conclusion

Our results show that K100, germacranolide 1-*β*,10-*α*-Epoxy-6*α*-hydroxy-1,10H-inunolide, exhibits a potent anti-inflammatory effect by inhibiting the ET-induced production of several markers of inflammation. Moreover, our data show that K100 is capable of binding to PTL protein targets at similar positions and with similar affinities. We further showed that K100 and PTL inhibit the proliferation, prolong of the S-phase of MDA-MB-231 cells and induce a shift in morphology of MDA-MB-231 cells from stellate to spherical in 3D cultures. This was associated with a decrease in the size of colonies. Both K100 and PTL significantly inhibit the invasion and motility of MDA-MB-231 cells. This study suggests a comparable effect for K100 and PTL on the tumor phenotype of MDA-MB-231 cells. Therefore, K100 can be perceived as a promising anti-inflammatory and antitumor molecule and its bioactivity warrants further investigation both* in vitro* and* in vivo*.

## 4. Experimental Section

### 4.1. Plant Material

The aerial parts of Lebanese* Cota palaestina* subsp.* syriaca* (Compositae) [2] were collected during the flowering season of the plant in the months of April and May 2002–2010. The plant material was identified by Dr. Stephen Jury at the University of Reading, UK. A voucher specimen has been deposited in the Post Herbarium (BEI) at the American University of Beirut. The aerial parts were dried by leaving the plant sample in the shade for two weeks before grinding it into around 10 mm pieces using a blender. The ground sample was subjected to decoction and/or methanol extraction.

### 4.2. K100 Extraction Procedure

The aerial parts of the plant were collected from Sahl El Jarmak, Lebanon, at an altitude of 1200–1800 m above sea level, in the flowering season of the plant in April and May, and processed for extraction. The plant shoots were left to air-dry for a week in the shade. When fully dried, the plant material was ground and sealed in vacuum bags.

Extracts from the plant (200 g) were prepared as previously described [17]. Briefly, the aerial parts were soaked, separately, in 2 L methanol for 16 h at room temperature. The crude methanolic extract “I” was concentrated to 1/10 of the volume and acidified to pH = 2 with a sulfuric acid solution. Alternatively, as recommended in herbal remedies, air-dried ground flower heads, stems, and leaves of* Cota palaestina* (4 mm square mesh) were soaked in boiling water (decoction, 1 : 8 w/v) for 30 minutes. The resulting crude-water extract “II” was filtered in 3 mm Whatman filter, then sterilized using the 0.2 *μ*m nonpyrogenic sterile-R filter, and stored at −20°C until used. Liquid-liquid extraction using a mixture of chloroform (CHCl_3_) : water (2 : 1 v/v) followed and the organic layer “I.2 or II.2” was collected and evaporated under reduced pressure at 40°C. I.2 or II.2 was applied to a liquid column chromatography (silica gel 0.035–0.075 mm, 60 Å, 1 kg) and fractionated using a gradient elution of petroleum ether (PE) : chloroform (CHCl_3_) : ethyl acetate (EtoAc) (2 : 2 : 1), PE : CHCl_3_ : EtoAc (1 : 2 : 1), CHCl_3_ : MeOH (3 : 1), and methanol. Ten subfractions of each I.2 and II.2 were collected and were concentrated* in vacuum* at 40°C. The residues were weighed, dissolved in ethanol, and assayed for their growth inhibitory activities against a panel of cell lines. The fractions containing sesquiterpene lactones were purified to isolate the bioactive molecules using solid phase extraction (SPE). The structure of the bioactive sesquiterpene lactone was elucidated using several spectroscopic techniques (1D NMR, 2D NMR, IR, HPLC-MS, and GC-MS). The spectroscopic data acquired for the pure bioactive compound were as follows: colorless; IR: 3468 (OH stretch), 2961, 2924, and 2854 (sp3 CH stretch), 1763 (C=O stretching of *α*,*β*-unsaturated-*γ*-lactone), 1616 (C=C stretch), and 1261 (CO stretch); 1H NMR (300 MHz, CDCl3) *δ* ppm: 6.44, 6.16 (each 1H, dd, H13), 5.34 (1H, brd, H5), 4.43 (1H, brd, H6), 4.11 (1H, dd, H8), 2.90 (1H, brd, H7), 2.68 (1H, brd, H1), 2.35 (2H, brd, H3), 2.23 (1H, s, OH, exchanges with D2O), 2.10 (1H, dt, H9a), 1.77 (3H, s, H15), 1.60 (1H, m, H9b), and 1.51 (3H, s, H14); EIMS, probe (70 ev) m/z (rel int.): (C15H20O4), 246 [M-H2O], 217, 185, 147, 105, 91, and 67.

The isolation of the bioactive molecule proceeded via “bioassay-guided fractionation.” Different concentrations of decoction extracts (decoction extracts/media; vol/vol), as indicated in respective experiments, were added to the cells 30 minutes prior to ET treatment.

### 4.3. Cell Culture

Low passage number (20–35) of SCp2 mouse mammary epithelial cells, subclones of CID-9 cells [17, 52] were seeded at 250 × 10^3^ cells/well using 6-well tissue-culture plates in Dulbecco's Modified Eagles Medium Nutrient Mixture F12 Ham (DMEM/F12) with 5% heat-inactivated fetal bovine serum (FBS), insulin (5 *μ*g/mL), and gentamycin (50 *μ*g/mL) in a humidified incubator (95% air, 5% CO2) at 37°C. Twenty-four hours after plating, cells were washed three times with phosphate-buffered saline (PBS) and FBS-free DMEM/F12 media containing insulin (5 *μ*g/mL) and incubated for 24 hours. Cells were then treated in 0% FBS media with whole plant extracts or with their fractions. Thirty minutes after treatment with plant extracts, ET was added to the cells in culture at 10 *μ*g/mL, except for control cells. Samples were collected 9–24 hours after ET treatment.

MDA-MB-231 human mammary adenocarcinoma cells were grown as described earlier [31]. For 2D cultures, MDA-MB-231 cells were plated in 24-well tissue-culture plates at a density of 5 × 10^4^ cells in each well. Living and dead cells were counted using the trypan blue dye exclusion assay after 24, 48, 72, and 96 hrs. For three-dimensional cultures, MDA-MB-231 cells were plated on the growth factor reduced matrigel obtained from BD Biosciences (BD number 354230) in 24-well tissue-culture plates at a density of 25 × 10^3^ cells in each well. The cells were maintained for 8 days before trypan blue counting.

Colony morphology was quantified by counting the number of spherical versus stellate colonies from phase contrast images having equal numbers of colonies. A colony was considered stellate if it had two or more extensions from the central colony of cells.

Parthenolide (BIOMOL-ENZO T-113-0050) prepared in dimethyl sulfoxide (DMSO) and K100 prepared in 98% ethyl alcohol both were stored in the dark at −20°C. In cell culture assays, drug working concentrations contained, as controls did, less than 0.1% DMSO and less than 0.5% ethyl alcohol in parthenolide and K100-treated cells, respectively.

### 4.4. Immunoassay of Interleukin-6

To measure IL-6 levels in conditioned media in response to ET in SCp2 cells, media collected 24 hrs after ET treatment were assayed by enzyme linked immunosorbent assay (ELISA) for IL-6 (Max Deluxe set mouse IL-6, BioLegend) according to the manufacturer's protocol and as described earlier [53].

### 4.5. Griess Reaction Assay of Nitric Oxide (NO)

NO levels in conditioned media were measured by the Griess assay for nitrite using a colorimetric Griess Reagent Kit (Invitrogen) as per the manufacturer's instruction and as described earlier [53].

### 4.6. SDS-Substrate Gel Electrophoresis (Zymography)

Gelatinase activity in conditioned media of ET-treated SCP2 cells was assayed by zymography as described earlier [54]. The activity of gelatinases was visualized as clear white bands on darkly stained blue gels.

### 4.7. Molecular Docking

The possibility of binding, precise location of binding sites, and predicted affinity of binding for each ligand was carried out using an automated docking software, Molegro Virtual Docker (MVD) 2010, version 4.2 (Molegro ApS, Aarhus, Denmark, http://molegro.com/), which is based on guided differential evolution and a force field based screening function [55]. The entire protein structures were obtained from the Protein Data Bank (PDB) and were loaded on the MVD platform for docking simulations, together with PDB files of K100, PTL, and DMAPTL. Multiple runs were done for each ligand and the results shown represent the average of five independent simulations.

### 4.8. Cell Cycle Analysis

Cells were trypsinized at 70% confluency for 2D cultures and at day 8 for 3D cultures were collected by centrifugation of 208 ×g for 5 min at 4°C and were fixed in ice-cold 70% ethanol for a minimum of 2 hrs and a maximum of 2 days. Cells were then centrifuged (208 ×g, 5 min, 4°C) and the pellet was washed with 1x PBS. DNase free RNase A was added at a concentration of 0.2 mg/mL (50 *μ*L) and cells were kept at 37°C in a water bath for 1 hr and 30 minutes to allow full digestion of RNA. The pellet was washed twice in 1x PBS before final resuspension in 420 *μ*L of 1x PBS into flow tubes (BD Falcon, USA). Thirty *μ*L of 2 mg/mL propidium iodide was then added to each flow tube and the cells were then analyzed using the flow cytometer, FACScan (Becton Dickinson, San Jose, CA).

### 4.9. Invasion/Motility Assay

Six-well tissue-culture plates were fitted with inserts (8 *μ*m pore size) coated with growth factor reduced EHS (matrigel). For the invasion assay [31], endothelial cells (ECV304) were grown in the inserts to confluency and then seeded with calcein-labeled tumor cells. After 24 hrs of coculture, the cells were treated for 16 hrs with K100 or PTL. Inserts were then removed and the endothelial cell layer was gently removed using a cotton swab. Fluorescent tumor cells that invaded through the endothelial layer were counted. A similar procedure was followed for the motility assay [31], except that the labeled MDA-MB-231 cells were seeded on the EHS-coated inserts directly, and after 24 hrs the cells were treated for 16 hrs with K100 and PTL. The inserts were then fixed using 4% formaldehyde in PBS for 20 minutes at room temperature. The cells towards the inside of the insert were removed using a cotton swab, and nuclei of migrated cells were counterstained with Hoechst (DAPI, 4,6-diamino-2-phenylindole) (Molecular Probes, Eugene, OR, USA) at a concentration of 0.5 *μ*g/mL, for 10 minutes at room temperature. The inserts were then examined by fluorescence microscopy. Cells that successfully moved through the 8 *μ*m pores were counted (10 random fields at 20x magnification) and were then plotted on a histogram as percentage of motile cells relative to the control cells.

### 4.10. Statistical Analysis

All experiments were repeated at least three times; statistical significance was determined using one-way ANOVA, unless otherwise indicated. *p* values less than 0.05 were considered statistically significant.
